# The newt (*Cynops pyrrhogaster*) RPE65 promoter: molecular cloning, characterization and functional analysis

**DOI:** 10.1007/s11248-014-9857-1

**Published:** 2014-12-10

**Authors:** Martin Miguel Casco-Robles, Tomoya Miura, Chikafumi Chiba

**Affiliations:** 1 Department of Life and Environmental Sciences, University of Tsukuba, Tennoudai 1-1-1, Tsukuba, Ibaraki 305-8572 Japan; 2 Graduate School of Life and Environmental Sciences, University of Tsukuba, Tennoudai 1-1-1, Tsukuba, Ibaraki 305-8572 Japan; 3 Faculty of Life and Environmental Sciences, University of Tsukuba, Tennoudai 1-1-1, Tsukuba, Ibaraki 305-8572 Japan

**Keywords:** Newt, RPE65, Retinal pigment epithelium, Transgenesis

## Abstract

The adult newt has the ability to regenerate the neural retina following injury, a process achieved primarily by the retinal pigment epithelium (RPE). To deliver exogenous genes to the RPE for genetic manipulation of regenerative events, we isolated the newt RPE65 promoter region by genome walking. First, we cloned the 2.8 kb RPE65 promoter from the newt, *Cynops pyrrhogaster*. Sequence analysis revealed several conserved regulatory elements described previously in mouse and human RPE65 promoters. Second, having previously established an *I*-*Sce*I-mediated transgenic protocol for the newt, we used it here to examine the −657 bp proximal promoter of RPE65. The promoter assay used with F0 transgenic newts confirmed transgene expression of mCherry fluorescent protein in the RPE. Using bioinformatic tools and the TRANSFAC database, we identified a 340 bp CpG island located between −635 and −296 bp in the promoter; this region contains response elements for the microphthalmia-associated transcription factor known as MITF (CACGTG, CATGTG), and E-boxes (CANNTG). Sex-determining region box 9 (or SOX9) response element previously reported in the regulation of RPE genes (including RPE65) was also identified in the newt RPE65 promoter. Third, we identified DNA motif boxes in the newt RPE65 promoter that are conserved among other vertebrates. The newt RPE65 promoter is an invaluable tool for site-specific delivery of exogenous genes or genetic manipulation systems for the study of retinal regeneration in this animal.

**Electronic supplementary material:**

The online version of this article (doi:10.1007/s11248-014-9857-1) contains supplementary material, which is available to authorized users.

## Introduction

The retinal pigment epithelium (RPE) is a monolayer of neuroepithelium-derived cells located between the choroid and photoreceptors of the eye (Kennedy et al. 1998; Esumi et al. 2007). The RPE performs several key functions in vision: these include secretion, phagocytosis, epithelial transport, light absorption, as well as being involved in the visual cycle, forming part of the blood retinal barrier, and maintaining photoreceptor nourishment (Kennedy et al. 1998; Strauss 2005; Matsuda et al. 2014). During eye development, at the optic vesicle stage, formation of the presumptive RPE is determined by the microphthalmia-associated transcription factor (MITF) and orthodenticle homeobox 2 (OTX2) (Baumer et al. 2003; Hallsson et al. 2004; Pogenberg et al. 2012; Masuda and Esumi 2010). The RPE is associated with several diseases including age-related macular degeneration (Khandhadia et al. 2012) and proliferative vitreoretinopathy, both of which lead to vision loss (Chiba 2014).

In the adult newt, which is a urodele amphibian, the RPE has an additional function: specifically, the ability to regenerate the neural retina upon injury (Mitashov 1996; Cheon et al. 1998; Grigoryan et al. 1998; Tsonis and Del Rio-Tsonis 2004; Chiba et al. 2006; Beddaoui et al. 2012; Mizuno et al. 2012; Chiba 2014; Islam et al. 2014). At present, it is impossible to manipulate gene function in vivo in the RPE or RPE-derived cells of the newt. Therefore, RPE-specific transgene expression is required to perform a number of applications, particularly functional gene analysis to examine newt retinal regeneration. The tamoxifen inducible CreERT2-loxP site-specific recombination system, the short hair-pin RNA interference, and the RNA-guided CRISPR-Cas9 system are genetic tools that have not yet been exploited for studying the RPE retinal regenerative system of the newt.

The retinal pigment epithelium-specific 65 kDa protein (RPE65), also known as retinoid isomerohydrolase, is functionally conserved among vertebrates, and is commonly used as an RPE marker (Hamel et al. 1994; Aguirre et al. 1998; Golczak et al. 2010; Chiba et al. 2006; Kiser and Palczewski 2010; Matsuda et al. 2014). Alternatively, the promoter region of the VMD2 gene (Masuda and Esumi 2010), which encodes the RPE-specific marker protein bestrophin-1 (BEST1), has been used to generate RPE-specific transgenic mice (Iacvelli et al. 2011). BEST1 protein expression has not been examined in the newt. However, we previously characterized RPE65 protein expression in this amphibian (Chiba et al. 2006), and sought to identify its RPE65 promoter for use in the present study. In mice, the RPE65 promoter has been shown to drive site-specific transgene expression in RPE cells (Boulanger et al. 2000; Boulanger and Redmond 2002). Similarly, to drive transgene expression in the newt RPE, we cloned and characterized the 2.8 kb upstream region of its RPE65 gene. Here, we show that the newt RPE65 upstream region contains a functional −657 bp proximal promoter capable of driving transgene expression in the RPE of F0 transgenic newts.

## Materials and methods

All animals in these experiments were cared for according to the University of Tsukuba Animal Use and Care Committee (AUCC) guidelines.

### Newts

Sexually mature *Cynops pyrrhogaster* were obtained from Toride–Imori (http://imori-net.org/) as described in Islam et al. (2014) and Nakamura et al. (2014). Adult newts were kept in polyethylene containers in water at 18 °C under normal day/night light cycles (Casco-Robles et al. 2010) until the transgenic experiments started.

### Isolation of the newt cpRPE65 promoter region

Newt genomic DNA was extracted from tail tips using a Wizard Genomic DNA Purification Kit (Promega, Madison, WI, USA). The cpRPE65 promoter was isolated using a Universal Genome Walker Kit (Clontech, Mountain View, CA, USA). This kit was modified for the newt genome by adding an additional restriction enzyme library *Hpa*I (New England Biolabs, Ipswich, MA, USA) and optimizing primer concentrations, as mentioned below. Adaptors were ligated according to the kit instructions. PCR was carried out using an Advantage GC 2 Kit (Clontech); this kit relaxes DNA secondary structures and improves amplification of GC-rich areas found in the regulatory regions of promoters. Reverse gene-specific primers were designed from the newt RPE65 mRNA exon 1 (DDBJ accession no. AB095018.1). Gene-specific reverse primer 1 (TGCTCGACATTCTGGCGTGCATGGAGAGTG) and gene-specific reverse primer 2 (GCCTCCGGCAGGTCCCACTTCAGCATGC) were used. AP1 and AP2 primers (supplied by the kit) were each diluted to a final concentration of 0.1 μM and gene-specific primers were kept at 0.2 μM. The cycling parameters for touch-down PCR comprised five cycles at 95 °C for 20 s and 70 °C for 3 min, followed by 35 cycles at 95 °C for 20 s and 68 °C for 3 min, with a final 10 min extension at 70 °C. A 2.8 kb PCR product obtained from the *Hpa*I library was subcloned into a pCR2.1 TOPO TA cloning vector (Life Technologies, Carlsbad, CA, USA), transformed into Stbl3 cells (Invitrogen, Carlsbad, CA, USA), and then cultured at 30 °C. Plasmid DNA containing the RPE65 promoter region was isolated with a Plasmid Mini Prep Kit (Qiagen, Tokyo, Japan). The 2.8 kb region of the RPE65 promoter was sequenced using a Big Dye Terminator kit (Applied Biosystems, Austin, TX, USA) on a 3130 Genetic Analyzer (Applied Biosystems).

### Bioinformatic analyses

The 2.8 kb sequence of the newt RPE65 promoter was scanned for CpG islands using EMBOSS Cpgplot (http://www.ebi.ac.uk/Tools/seqstats/emboss_cpgplot/). MEME motif search was used to identify conserved motif consensus boxes (Bailey and Elkan 1994) using the online tool available at http://meme.nbcr.net/meme/cgi-bin/meme.cgi. TOMTOM (version 4.9.1) was used for identifying motif transcription factor similarity (Gupta et al. 2007); available at http://meme.nbcr.net/meme/cgi-bin/tomtom.cgi. Pattern search for transcription factor binding sites (PATCH) version 1.0 was used to identify transcription factor binding sites in the TRANSFAC 6.0 database (http://www.gene-regulation.com/cgi-bin/pub/programs/patch/bin/patch.cgi).

### Construction of the cpRPE65-mcherry01 reporter

The region controlling expression of the enhanced green fluorescent protein (EGFP) in the pCAGG-EGFP *I*-*Sce*I construct described previously (Sobkow et al. 2006; Casco-Robles et al. 2011) was modified by replacing EGFP with mCherry using *Bam*HI and *Bsp*1407I sites, yielding pCAGG-mCherry. Chicken HS4 2X core insulators were used to reduce any positional effect in the newt (Miura, unpublished data). A 5′ adaptor containing *Xho*I-*I*-*Sce*I-HS4 [2X]-*Bst*XI was amplified from pNI-CD (the chicken HS4 2X core insulator was a kind gift from Gary Felsenfeld at the National Institutes of Health, Bethesda, MD, USA) using an Advantage 2 PCR Kit (Clontech) with a forward primer containing *Xho*I and *I*-*Sce*I sites (aaactcgagTAGGGATAACAGGGTAATTAGGGCGAATTGGGCCCTCT) and a reverse primer containing a *Bst*XI site (aaaccaccgcggtggTAGAATACTCAAGCTATGCA). The 3′ adaptor *Afl*II-HS4 [2x]-*I*-*Sce*I–*Dra*III was amplified using a forward primer containing an *Afl*II site (aaacttaagTAGGGCGAATTGGGCCCTCT) and a reverse primer containing an *I*-*Sce*I–*Dra*III sites (aaacacgtagtgATTACCCTGTTATCCCTATAGAATACTCAAGCTATGCA). The cycling parameters for both adaptors were 95 °C for 1 min, 35 cycles at 95 °C for 30 s and 68 °C for 1 min. PCR products were subcloned into pCR2.1 TOPO (Life Technologies), transformed into TOP10 cells (Life Technologies), cultured at 37 °C, and the plasmid DNA was extracted using a Plasmid Mini Prep kit (Qiagen). Adaptors were released from pCR2.1 by digestion with *Xho*I/*Bst*XI or *Afl*II/*Dra*III. The 5′ *I*-*Sce*I-HS4 [2x] and the 3′ HS4 [2X]-*ISce*I adaptors were introduced into the *Xho*I/*Bst*XI or *Afl*II/*Dra*III sites of pCAGG′-mCherry. The CAGG promoter was released from the construct by digestion with *Bst*XI and *Kpn*I. The RPE65 newt promoter comprising −657 bp to +28 bp was amplified by the Advantage 2 PCR kit (Clontech) using the following primers: *Bst*XI-RPE65_FP: gagtatttctaccacAAGTCGGATTACAGTTTTTATGTCT and RPE65_*Kpn*I_RP: gatcccgggcccgcgGCCTCCGGCAGGTCCCACTTCAGC. The cycling parameters were 95 °C for 1 min, 30 cycles at 95 °C for 30 s, 63 °C for 30 s, and 68 °C for 1 min. The PCR product was inserted between the *Bst*XI and *Kpn*I sites using an In-Fusion HD cloning kit (Clontech). This construct was transformed into Stbl3 cells (Invitrogen), cultured at 30 °C, and the plasmid DNA was isolated as described above. For simplicity, *I*-*Sce*I-*2XHS4*-*cpRPE65*-*mCherry*-*pA*-*2XHS4*-*I*-*Sce*I is now referred to as cpRPE65-mcherry01.

### Promoter analysis in transgenic newts

One-cell stage fertilized embryos were obtained using a semi-natural two-tank mating system (Casco-Robles et al. 2010). Transgenic newts containing cpRPE65-mcherry01 were generated and reared according to a transgenic newt protocol (Casco-Robles et al. 2011). Briefly, *I*-*Sce*I recognition sites (5′-TAGGGATAACAGGGTAAT-3′) in the transgene construct are targeted by co-microinjection of *I*-*Sce*I enzyme (a rare cutter). This co-microinjection of *I*-*Sce*I and transgene DNA significantly improves transgene insertion into the genome (Thermes et al. 2002; Pan et al. 2006; Sobkow et al. 2006; Casco-Robles et al. 2010; Bevacqua et al. 2013). For the cpRPE65-mcherry01 construct, 40–160 pg of DNA and 0.001 units of *I*-*Sce*I enzyme were co-microinjected at the one cell-stage of *C. pyrrhogaster*. A group of non-injected one-cell stage embryos were set aside and kept as a viability control. F0 transgenic newts were reared until stage 59 and monitored for promoter activity during their developmental stages, according to the newt standard development table (Okada and Ichikawa 1947). Bright light and fluorescence images were taken using a digital camera (C-5060; Olympus, Shinjuku, Tokyo, Japan) attached to a fluorescence stereomicroscope (Leica M165 FC, Exton, PA, USA) with a filter set for mCherry (Leica). The presence of cpRPE65-mcherry01 was detected in the F0 transgenic larvae (stage 59) by extracting genomic DNA from their tail tips. DNA was amplified using a Kod FX PCR kit (Toyobo, Japan) with the cpRPE65 forward primer (AAGTCGGATTACAGTTTTTATGTCT) and mCherry reverse 5′ primer (CATGTTATCCTCCTCGCCCTTGC). The cycling parameters comprised 32 cycles at 98 °C for 10 s, 61 °C for 30 s, and 68 °C for 1 min.

### Immunohistochemistry

At stage 59 (swimming larvae just prior to metamorphosis), the transgenic animals were administered anesthesia in the form of 0.05 % (v/v) FA100 (4-allyl-2-methoxyphenol; DS Pharma Animal Health, Japan) and then sacrificed. Tissues were fixed with 4 % (w/v) paraformaldehyde (Wako, Japan), 0.25 % (v/v) glutaraldehyde (Wako), 1× phosphate-buffered saline (PBS) pH 7.4, for 6 h at room temperature. Cryosections of the eye were generated on a cold tome and prepared using standard immunohistochemistry techniques. Bright light and fluorescence images of sections were taken with or without the mCherry filter (Leica) mounted on a brightfield microscope (BX50, Olympus, Shinjuku, Tokyo, Japan) using a charge-coupled device camera (C4742-95 ORCA-ER system, Hamamatsu Photonics, Japan). To confirm mCherry expression in the RPE, sections were treated with a 1:1,000 dilution of a rabbit dsRed primary polyclonal antibody (Clontech), followed by a 1:1,000 dilution of a goat secondary anti-rabbit IgG conjugated antibody (Vector Labs, Burlingame, CA, USA). Sections were blocked using components of an ABC Blocking Kit (Vector Labs) and treated thereafter with an immunoreactivity DAB substrate (Vector Labs). Negative control sections were not treated with the primary antibody. Pigmentation was removed from sections by bleaching the tissue with 1.5 % (v/v) sodium azide (Wako) and 15 % (v/v) hydrogen peroxide (Wako) in 1× PBS overnight.

### Digital illustrations

Fluorescence, bright-light image contrast, brightness and sharpness were adjusted using Photoshop CS5 (Adobe, San Jose, CA, USA). Figures were prepared using Illustrator CS5 graphics software (Adobe).

## Results and discussion

### Characterization of the newt RPE65 upstream region

**Fig. 1:**
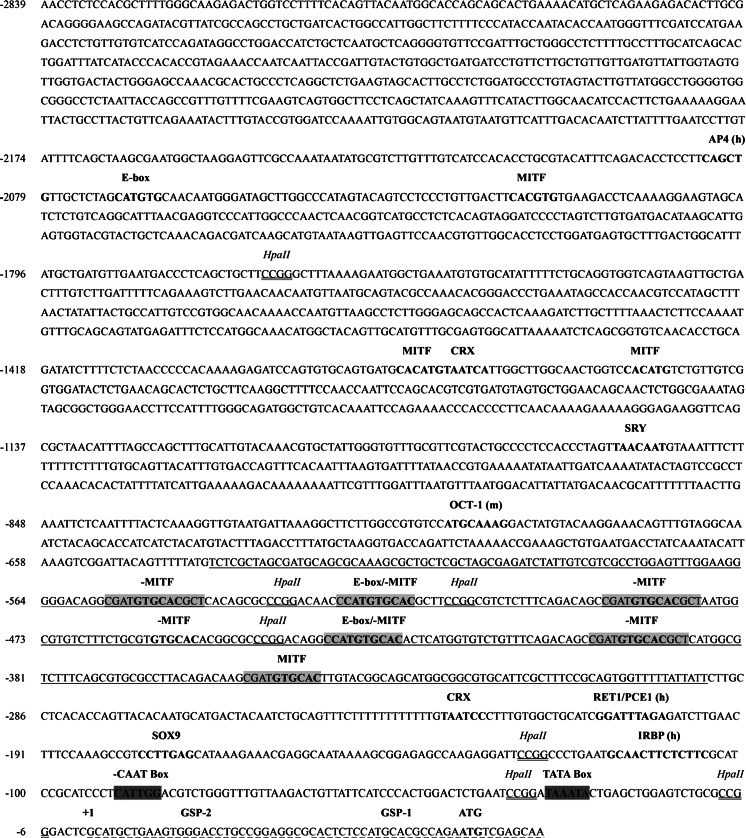
Nucleotide sequence of the *C. pyrrhogaster* RPE65 upstream region. Nucleotide numbers (relevant) appear on the *left-hand side*. CpG island is *underlined*. *Hpa*II sites are *double-underlined in italics*. Repeat regions from newt are shown in *gray boxes*. Human and mouse RPE65 promoter-derived elements (Nicoletti et al. 1998; Boulanger et al. 2000) are shown in *bold with parenthesis* (***h***) and (***m***), respectively. GSP1 and GSP2 reverse primers for genome walking are shown in *dashed-underlined bold font*. SOX9, SRY, CRX, E-box, and MITF response elements are shown in *bold*. CAAT and TATA *boxes* are *highlighted in green*. *Inverted elements* on the *minus* strand are indicated with a (-). (Color figure online)

The large genome size of newts and salamanders has hindered the isolation of promoter regions in these amphibians; indeed, *C. pyrrhogaster* has a C-value of 37.8 (Licht and Lowcock 1991). Here, we applied a modified genome walking strategy to extract a 2.8 kb upstream region of the newt RPE65 gene, the sequence of which is available in GenBank (newt cpRPE65; accession no. KM099425). The RPE65 upstream sequence from newt, including its characterized elements and previously identified human RPE65 (Nicoletti et al. 1998) and mouse RPE65 (Boulanger et al. 2000) promoter elements, is shown in Fig. 1. Using the TRANSFAC database, we identified a TATA box (TAAATA) between nucleotide positions −33 to −27; this box is common to newt Prod1 and lens promoters (Ueda et al. 2005; Shaikh et al. 2011). In addition, an inverted CAATG box was found at positions −90 to −85, upstream of the TATA box. Human light-response elements such as the photoreceptor conserved element 1 (known as RET1/PCE1) and the interphotoreceptor retinoid-binding protein (known as IRBP), which are characterized as eye specific (Kimura et al. 2000; Cunningham and Gonzalez-Fernandez 2003; Gonzalez-Fernandez et al. 2009), are located in the newt promoter at sites −210 to −202 and −117 to −106, respectively. An OCT-1 (POU2F1) box ATGCAAAG motif reported by (Boulanger et al. 2000; Tantin et al. 2005) is located in the newt promoter at −793 to −786. Two cone-rod homeoboxes (CRX) TAATC[C/A], SOX9 CCTTGAG, and SRY [A/T]AACAA[A/T] response sites are located at −1,364 to −1,359, −231 to −226, −178 to 172 and −1,059 to −1,053, respectively. CRX and SOX9 are both expressed in the RPE (Esumi et al. 2009; Matsuda et al. 2014). A CpG plot revealed an *Hpa*II site-containing 340 bp CpG island, located between −635 and −296 bp in the newt proximal promoter. *Hpa*II restriction sites have recently been used as predictive tools for detecting CpG islands (Barrera and Peinado 2012). Additionally, MEME scan analysis detected six unique repeat boxes within the 340 bp CpG island of the newt promoter; these boxes share a CSATGTGCAC consensus sequence and are located at positions −556 to −547, −526 to −517, −436 to −427, −491 to −482, −401 to −392, and −354 to −345.

### Distribution of MITF response elements and conserved motif sites

**Fig. 2:**
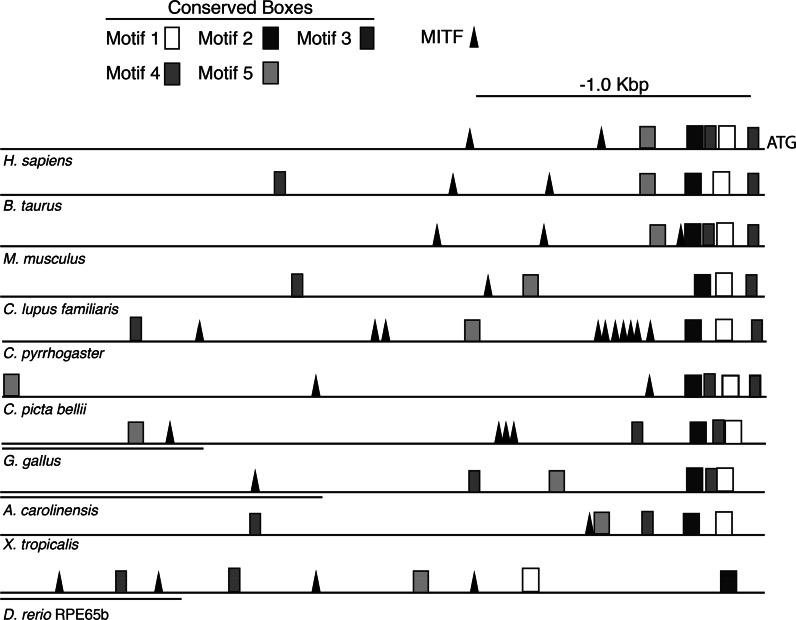
Schematic comparison of the RPE65 promoter among vertebrates. The vertebrate RPE65 upstream promoter regions (2.8 kb from ATG start codon) were analyzed by MEME pattern motif recognition and TRANSFAC 6.0 software. All vertebrates contained at least one MITF response site, a critical factor for RPE development. The consensus sequences for the motifs described herein are shown in Tables S 2–6 Online Resource. In most higher vertebrates, *motif boxes* 1, 2, and 3 have a conserved distribution proximal to the ATG codon. *Motif boxes* 1 and 2 contain nuclear factor 1 (NF-1) and activating enhancer binding protein 4 (AP-4); both are derived from mammalian promoters. Motif 2 was found to be rich in CRX elements. Motif 3 contains the 5′ UTR. NF-1, AP-4, and the 5′ UTR have been shown to be key elements for driving a luciferase reporter gene in mouse and human promoters (Boulanger et al. 2000; Nicoletti et al. 1998). Sequence sites for *motif boxes* 4 and 5 have not been previously characterized in mammalian promoters (Boulanger et al. 2000; Nicoletti et al. 1998). A TOMTOM motif scan revealed that motif 4 contains tentative elements with sequence similarity values of *p* < 0.01 for the neural retina leucine zipper (NRL), the sex-determining region Y box (SRY) and FOXO1. Motif 5 shares sequence similarity with LHX2 (*p* < 0.005). *Double underlining* denotes an annotated mRNA site upstream of the RPE65 promoter. The accession numbers corresponding to the above species are shown in Table S1 Online Resource. RPE65b is the fish (*D. rerio*) RPE65 ortholog previously described by Schonthaler et al. (2007)

PATCH running TRANSFAC 6.0 identified several MITF response elements in the newt promoter, the majority of which are located within the repeats sites (Fig. 1). MITF binds to the functional palindromic CACGTG (E-box), the non-palindromic CACATG (Levy et al. 2006) and the ATGCATGTG (M-box) (Aksan and Goding 1998; Goding 2000; Pogenberg et al. 2012). MITF is known as the master regulator of melanocyte development, is an oncogene in melanomas, and a key player in RPE development (Goding 2000; Levy et al. 2006; Hoek et al. 2008; Vetrini et al. 2004; Pogenberg et al. 2012). Although MITF elements are common in vertebrate RPE65 promoters, the distribution, locations and percentage of MITF response sites within a CpG island are unique to the newt RPE65 promoter (Fig. 2; Table S1 Online Resource). These sites should now be examined in further detail. Interestingly, other RPE genes are also targeted by MITF; these include BEST1 (Esumi et al. 2007), tyrosinase (Shihabara et al. 2000), and SLC11A1 (Hoek et al. 2008). The BEST1 promoter is regulated by SOX9 interactions with MITF and OTX2 (Masuda and Esumi 2010) and its proximal promoter (positions −253 to +38) contains two MITF E-boxes shown to be sufficient to drive transgene expression in the RPE (Esumi et al. 2007). Our MEME analysis of the newt RPE65 promoter region revealed several motif boxes, the positions of which are conserved among vertebrate RPE65 promoters (Fig. 2; Tables S 2–6 Online Resource). SRY and LHX2 motifs were both identified as tentative sites for the uncharacterized vertebrate motif boxes 4 and 5 (Fig. 2; Tables S 2–6 Online Resource). SOX9 (SRY sites) and LHX2 also participate in the regulation of genes involved in the visual cycle of the RPE, including the regulation of RPE65 (Matsuda et al. 2014).

### Functional promoter assays in F0 transgenic newts

**Fig. 3:**
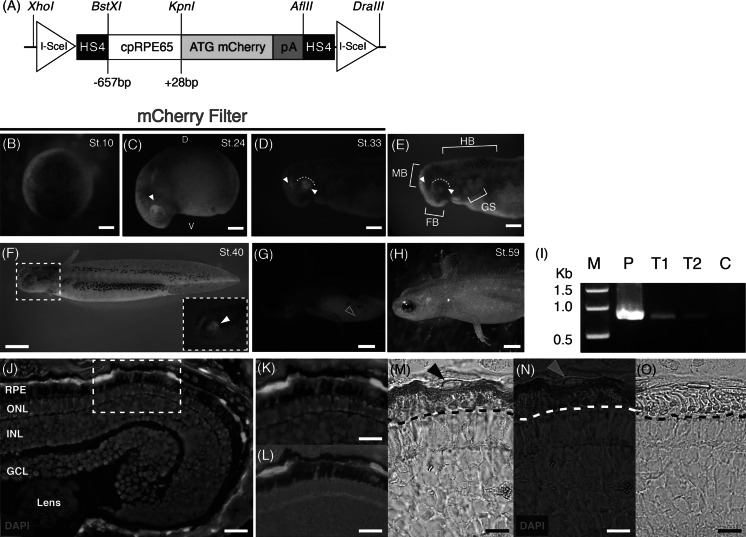
RPE65 proximal promoter (−657 bp) assay in F0 transgenic newt larvae (C. pyrrhogaster). **a** Schematic representation of the transgene construct. **b** No detection of mCherry expression at stage 10 (blastula). **c** Stage 24 (early tailbud) onset of mCherry expression localized in the developing optic cup of the dorsal posterior margin, indicated with a *white arrowhead*. *D* dorsal, *V* ventral. **d** Stage 33 (tailbud) showing mCherry expression in the midbrain and posterior eye margin. **e** Bright field image of (**d)**. *FB* forebrain, *MB* midbrain, *HB* hindbrain, *GS* gill slits. (**d**, **e**) *White arrowheads* denote mCherry, and the *white dotted* arcs indicate the posterior eye margin. **f** Stage 40 (early swimming larva) showing induction of pigmentation in the eye. *Inset* (*dashed White Square*) shows a magnified image of the developing eye expressing mCherry denoted by the *white arrowhead* (**g)**. **h** Stage 59 (mature larva) showing a heavily pigmented eye. *Yellow* *arrowhead* indicates auto-fluorescence. **i** PCR detection of cpRPE65-mCherry01 in genomic DNA from stage 59 F0 transgenic larvae. *P* Positive control cpRPE65-mCherry01 plasmid; *T1* transgenic larva genomic DNA sample 1; *T2* transgenic larva genomic DNA sample 2; *C* Negative control wild-type genomic DNA. **j** Promoter activity in the retinal pigment epithelium (*RPE*). *ONL* outer nuclear layer; *INL* inner nuclear layer; *GCL* ganglion cell layer. **k** Magnification of dashed *white rectangle* in (**j)**. **l** Magnification of dashed white rectangle in (**j)**, showing mCherry/bright field merged image of RPE apical microvilli. **m, n** Immunostaining of a retinal section with an anti-mCherry antibody. Signal was visualized by DAB treatment (*brown*). *Black* and *yellow arrowheads* indicate the RPE cell nucleus. **o** Negative control (without anti-mCherry antibody). *Dashed black* and *white lines* in (**m**, **n**, **o)** indicate the apical microvilli of the RPE. *Scale bars*, **b–e** 0.5 mm, **f** 2.0 mm, **g–h** 0.4 cm, **j–o** 50 μm. (Color figure online)

To verify if the newt RPE65 proximal promoter (−657 to +28), which contains a CpG island, MITF response elements and motif boxes 1–3, is capable of driving transgene expression in the RPE, we generated the reporter construct shown in Fig. 3a. Optimization of the microinjection conditions used to generate cpRPE65-mcherry01 F0 transgenic newts are shown in Table 1. Promoter activity was first detected at embryonic stage 24 when it was localized in the developing early optic cup (Fig. 3c). mCherry expression was maintained in the dorsal posterior margin during stage 33 (Fig. 3d). We previously detected RPE65 protein expression with immunohistochemistry at stage 32 prior to eye pigmentation (Islam et al. 2014) and also at stage 43 (Chiba et al. 2006). When pigmentation in the eye was present, RPE65 promoter activity expanded towards the center of the eye (Fig. 3f). By stage 59, the eye was heavily pigmented and mCherry fluorescence was difficult to detect (Fig. 3g, h); therefore, eye sections were prepared to counteract this issue. Proximal promoter activity was detected in the RPE of the mature eye (Fig. 3j–n; Table 1). The role of OCT-1 (−793 to −786) in the RPE65 promoter remains unclear. OCT-1 is unlikely to participate in RPE specificity, as was noted in the mouse (Boulanger et al. 2000); furthermore, it is not required for transgene expression in the newt RPE. Conserved motif boxes 4 and 5 were also redundant in terms of driving transgene expression in the newt RPE. Hence, the −657 to +28 promoter region must contain the key sites for transcription of RPE65 in the newt.

**Table 1:** Optimization of cpRPE65-mCherry01 F0 generation transgenic newts

DNA pg^a^/egg	Eggs injected^b^	Survival St.10 blastula (%)	mCherry expression					Survival^d^ St. 59 (%)
			Developing eye (%)			St. 59 RPE^c^		
			ND	Avg.	Strong	+	–	
160	120	22 (18)	8	11 (50)	3 (13.6)	4/5	1/5	9 (41)
100	112	45 (40)	32	9 (20)	4 (9)	3/3	0/3	23 (47)
80	103	68 (66)	60	6 (9)	2 (3)	2/2	0/2	46 (68)
40	108	77 (71)	74	3 (4)	0	2/2	0/2	64 (83)
0	–	64^e^ (97)	–	–	–	–	–	59 (92)

*ND* not detected, *St.* stage, *RPE* retinal pigment epithelium, *Avg.* average

^a^pg is the total mass of cpRPE65-mCherry01 including the vector backbone

^b^The injection volume was fixed at 2 nL/egg. *I*-*Sce*I enzyme concentration was fixed at 1× 10–3 U/egg

^c^A subset of the average mCherry-expressing embryos were reared until stage 59 and their eye sections were prepared as shown in Fig. 3j–o

^d^Based on surviving blastula embryos

^e^Viability control group (n = 66)

## Conclusion

Similar to its mammalian counter parts, the newt RPE65 proximal promoter is sufficient to drive expression in the RPE. This proximal promoter is beneficial to drive in vivo transgene expression in the RPE for practical genetic manipulation systems, a previous obstacle in the study of newt retinal regeneration. During retinal regeneration in adult newts, the proto-oncogenes FGF2, FGFR-1/2, MEK1/2, ERK1/2, Hes-1, Notch-1 and Musashi-1, along with retinal transcription factors and stem cell markers such as Pax6 and Chx10, are expressed in RPE-derived cells (Chiba et al. 2006; Nakamura and Chiba 2007; Susaki and Chiba 2007; Chiba and Mitashov 2007; Kaneko and Chiba 2009). Recently, (Islam et al. 2014) found that during retinal regeneration mature RPE cells can reprogram themselves to become multipotent (Mitf, Sox2, Klf4, c-Myc, and Pax6) while retaining RPE65 expression. Functional gene analysis is critical if we are to gain better understanding of the molecular mechanisms involved in retinal regeneration in newts. To examine potential loss-of-function effects for the above mentioned factors, we successfully knocked-down Pax-6 expression using shRNAi transgene constructs with the CAGG promoter and generated eyeless and small-eyed newts (Islam et al. 2014). Similarly, it is now possible to use the newt RPE65 promoter with shRNAi to knockdown Mitf, Sox2, Klf4, c-Myc and Pax6 in the RPE, which is the primary cell source for regeneration of the retina. Furthermore, the newt RPE65 promoter can be used to drive the RNA-guided CRISPR-Cas9 system, the CreERT2-loxP system for conditional activation targeting loss or gain of function in the RPE, or for RPE cell tracking, which are all invaluable tools in this field of research.

## Electronic supplementary material

Below is the link to the electronic supplementary material.

Supplementary material 1 (DOCX 181 kb)
