# Supplementary material for: The newt (Cynops pyrrhogaster) RPE65 promoter: molecular cloning, characterization and functional analysis
Source: Transgenic Res. 2014 Dec 10;24(3):463–73. doi: 10.1007/s11248-014-9857-1 (PMC4436847; doi:10.1007/s11248-014-9857-1)
Supplement: Supplementary file 1 — Supplementary material 1 (DOCX 181 kb) [file 11248_2014_9857_MOESM1_ESM.docx]

**Electronic Supplementary Material**

**The newt (*Cynops pyrrhogaster*) RPE65 promoter: molecular cloning, characterization and functional analysis**

**Martin Miguel Casco-Robles • Tomoya Miura • Chikafumi Chiba**

M. M. Casco-Robles (Corresponding author)

Department of Life and Environmental Sciences, University of Tsukuba, Tennoudai 1-1-1, Tsukuba, Ibaraki 305-8572, Japan Tel: + 81-090-9138-0659; Fax: +81-29-853-6614; email: casco.m.m.gm@u.tsukuba.ac.jp or martinmcasco@gmail.com

T. Miura

Graduate School of Life and Environmental Sciences, University of Tsukuba, Tennoudai 1-1-1, Tsukuba, Ibaraki 305-8572, Japan

C. Chiba (Corresponding author)

Faculty of Life and Environmental Sciences, University of Tsukuba, Tennoudai 1-1-1, Tsukuba, Ibaraki 305-8572, Japan Tel: + 81-29-853-4667; Fax: +81-29-853-6614; email: chichiba@biol.tsukuba.ac.jp

| Supplementary Table 1. Sequence comparison of CpG islands and MITF response elements in the RPE65 2.8 kb promoter region among vertebrates | | | | | | | |
| --- | --- | --- | --- | --- | --- | --- | --- |
|  |  |  | **Elements within a CpG island** | |  | | |
| Species | **MITF ^a^** | **CpG island^b^**  **(position)** | ***HpaII***  **CCGG** | **MITF ^c^**  **(%)** | | | **Accession no./**  **reference** |
| Newt  (*C. pyrrhogaster)* | 10 | 340bp  (-635 to -296) | 3 | 7 (70) | | KM099425 | |
| Dog  (*C. lupus familiaris)* | 1 | 447bp  (-368 to -27) | 7 | 0 | | NW_876321.1 | |
| Frog  (*X. tropicalis*) | 1 | 366bp  (-987 to -622) | 0 | 0 | | NW_003163757.1 | |
| Chicken  (*G. gallus*) | 4 | ND | - | - | | NC_006095.3 | |
| Fish  (*D. rerio* RPE65b) | 3 | ND | - | - | | NW_001879345.3 | |
| Mouse  (*M. musculus*) | 3 | ND | - | - | | AF271297.1, CM000211.2  Boulanger et al. 2000 | |
| Turtle  *(C. picta bellii)* | 2 | ND | - | - | | NC_024225.1 | |
| Human  (*H. sapiens*) | 2 | ND | - | - | | NG_008472.1  Nicoletti et al. 1998 | |
| Cow  (*B. taurus*) | 2 | ND | - | - | | NW_003103871.1 | |
| Lizard  (*A. carolinensis*) | 1 | ND | - | - | | NW_003338875.1 | |
| ^a^ Number of 5′ to 3′ MITF (CACGTG, GTGCAC, CACATG) response sites within the 2.8 kb region upstream of the ATG start codon.  ^b^ CpG plot search using default: Observed/Expected ratio > 0.60 and Percent C + Percent G > 50.0. ND: Not Detected.  ^c^ Number of MITF sites within the CpG island.  RPE65b: The fish *(D. rerio*) RPE65 ortholog previously described by Schonthaler et al. 2007. | | | | | | | |

| Supplementary Table 2. Conserved motif 1 description | | | |
| --- | --- | --- | --- |
| Motif 1:  IRBP/AP-4 | | | 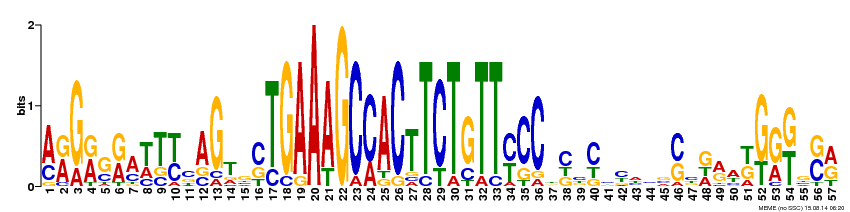 |
| RPE65 promoter | **Strand** | ***p*-value** | **Sequence** |
| *C. picta bellii* | + | 1.31e-22 | IRBP AP-4 NF-1  AGGAGGATTTCAGTGGTGAAAGCAACTTCTGTTCCCTGGTACAA**GCTGA**TTGGTTGG |
| *H. sapiens* | + | 2.70e-21 | AP-4 IRBP AP-4  AGGGGGATCTGAGA**GCTGA**AAGCAACTTCTGTTCCCCCTCCCTCA**GCTGA**AGGGGTG |
| *A. carolinensis* | + | 2.41e-18 | IRBP NF-1  AGGAGGATTTGAGTAGTGAAAGACACTTCTGTTTGCATGGCCAAGCTAATTGGTTCA |
| *B. taurus* | + | 2.70e-18 | AP-4 IRBP AP-4  AGAAGTATCTGAGT**GCTGA**AAGCCACTTCTGTTTTCCCTCTCTCA**GCTGA**GGAGGGT |
| *X. tropicalis* | + | 3.55e-17 | IRBP NF-1  AAGAGAATTCCACTTCTGAAAGCCACTTCTGTCTCGGCTCCTCGTGGGGATTGGCCA |
| *C. pyrrhogaster* | + | 1.70e-16 | IRBP**?** NF-1  AAGAGGATTCCGGCCCTGAATGCAACTTCTCTTCGCATCCGCATCCCTCATTGGACG |
| *C. lupus familiaris* | + | 1.23e-15 | AP-4  GCGGCACAGCCCGG**GCTGA**AAGCCGCTCCTGTTCCCGCCTGGGCCGGGGCGGGGAGG |
| *M. musculus* | + | 1.48e-14 | AP-4  CAAGCATCTAAAGTATTGAAAGCCACTTTTGTTACCTTCCATCA**GCTGA**GGGGTGGA |
| *G. gallus* | + | 2.15e-14 | NF-1  CGGGAGGATTTCGGCCTGAAAGCCACCTCTGTTCCAGCACTGAGCCCATTGGCTGCA |
| *D. rerio* RPE65b | - | 1.76e-11 | CAGGTACATTTGGTGTCCAAAGCCACATTTTTTCCCTGCTTTTTAAAAACATATGGT |
| Human-derived RPE65 regulatory elements are highlighted in yellow (Nicoletti et al. 1998).  Mouse-derived RPE65 regulatory elements are highlighted in bold (Boulanger et al. 2000).  Patch 1.0 running Transfac 6.0 (green).  The corresponding accession numbers for these sequences are shown in S. Table 1 Online Resource. | | | |

| Supplementary Table 3. Conserved motif 2 description | | | |
| --- | --- | --- | --- |
| Motif 2:  CRX sites  NF-1 half-site: TGGAN  Full: TGGAN(5)GCCA | | | 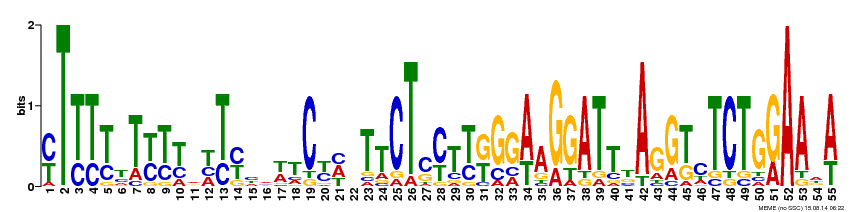 |
| RPE65 promoter | **Strand** | ***p*-value** | **Sites** |
| *H. sapiens* | + | 4.30e-26 | Crx Ret1/PCE1 NF-1  CTTTCTTTCTAATCTGTTCTCATTCTCCTTGGGAAGGATTGAGGTCTC**TGGAAAA** |
| *B. taurus* | + | 8.41e-23 | Crx Ret1/PCE1 NF-1  TTTCTTTCTAATCCACTTCTTGTTCTCTTTGGGAAGGATTGAGGTCTC**TGGAAGA** |
| *C. picta bellii* | + | 1.41e-20 | Crx Ret1/PCE1 NF-1  TTCTTGTTTCTTTGTAATCCATTTCTGCTTGGGAAGGAATTAGGTCTC**TGGAAAA** |
| *G. gallus* | + | 6.40e-17 | Crx  TTTCTTTTTTCTTTGTAATCTCTTCTGCTCGGGAAGAATCTAGGTCTCTGAAAAA |
| *C. lupus familiaris* | + | 2.45e-15 | CTCCCCAGCCCACCCGCCCGCCGTCTCCTCGGGAGGGATTGAGGTCTC**TGGAGGA** |
| *M. musculus* | + | 1.42e-13 | NF-1  TTTTTATCCAACTCACTACTCCCTCTCCCTTGAAAGGATCCAAGTC**TGGAAAA**TA |
| *C. pyrrhogaster* | + | 2.46e-13 | Crx Ret1/PCE1  CTTTTTTTTTTTTGTAATCCCTTTGTGGCTGCATCGGATTTAGAGATCTTGAACT |
| *X. tropicalis* | + | 3.19e-11 | Crx NF-1  CTTTTCTTTCGATTCATTCTTTTAATCCAGTCGTGGTTTGAAAGGATT**TGGAATT** |
| *A. carolinensis* | + | 4.53e-11 | Crx NF-1  CTTTTCCCTTGTTTCCTTGTAATCCACTTCTGCATGGAGATTTAAGTC**TGGAAAA** |
| *D. rerio* RPE65b | - | 1.89e-09 | ATCTCTTTGTGCTCAAACCAAGAACTACTTCACTAATGTTTACCTTCC**TGAAAGA** |
| Human-derived RPE65 regulatory elements are highlighted in yellow (Nicoletti et al. 1998).  Mouse-derived RPE65 regulatory elements are highlighted in bold (Boulanger et al. 2000).  Patch 1.0 running Transfac 6.0 (green).  The corresponding accession numbers for these sequences are shown in S. Table 1 Online Resource. | | | |

| Supplementary Table 4. Conserved motif 3 description | | | |
| --- | --- | --- | --- |
| Motif 3  5′UTR | | 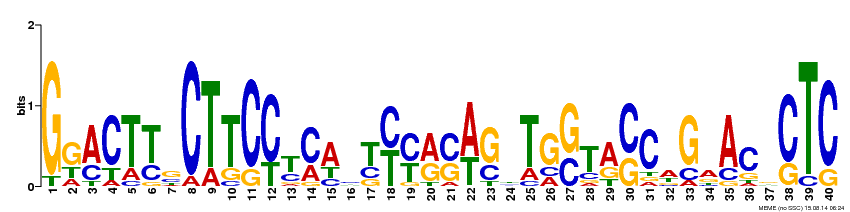 | |
| RPE65 promoter | **Strand** | ***p*-value** | **Sites** |
| *B. taurus* | + | 1.06e-21 | GGACTTGCTTCCTCAGTCCACAGTTGGTGCCAGAACTCTC |
| *M. musculus* | + | 3.33e-19 | GAACTTGCTTCCTCATCCTACAGCTGGTACCAGAACTCTC |
| *H. sapiens* | + | 5.01e-16 | GAACTTCCTTCTTCATTCTGCAGTTGGTGCCAGAACTCTG |
| *C. lupus familiaris* | + | 9.40e-14 | GGCCTCGCTGCCTCCGTCCGCTCGTGCTACCTCGACCGTC |
| *C. picta bellii* | + | 8.07e-11 | GTTCTGACTTCCACACTTCGCAGTTGGCTCTGGAAAGCTC |
| *A. carolinensis* | - | 5.00e-10 | GGATTTTCTTCTGAACTTTAGAGAAGGTACCAAGGTGCTG |
| *C. pyrrhogaster* | + | 8.50e-10 | GGACTCGCATGCTGAAGTGGGACCTGCCGGAGGCACACTC |
| *X. tropicalis* | - | 2.03e-09 | GTATTTTATTGCCATTCTTTGTCATCGTAGCAGTACGGTC |
| *G. gallus* | - | 2.57e-09 | GGCCCCCCTCCCCCCCCTCAGTGCCACATCCCCACGGCTC |
| *D. rerio* RPE65b | - | 5.63e-09 | TTAAATGCTGCTTATCGCCACATGTCGGAGCTGTAGCCTC |
| The corresponding accession numbers for these sequences are shown in S. Table 1 Online Resource. | | | |

| Supplementary Table 5. Conserved motif 4 description | | | |
| --- | --- | --- | --- |
| Motif 4  Uncharacterized  Tentative TF TOMTOM: p<0.01  FOXO1 (MA0480.1)  NRL (Neural Retina leucine zipper ) like  Sry (UP00016_2) Ref: Masuda et al. 2014 | |  | 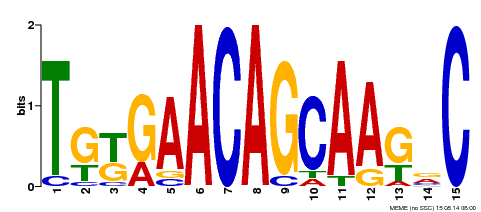 |
| RPE65 promoter | **Strand** | ***p*-value** | **Sites** |
| *G. gallus* | + | 2.22e-09 | TGTGAACAGCAAGAC |
| *H. sapiens* | + | 4.22e-09 | TGGGAACAGCAAGCC |
| *C. picta bellii* | + | 1.22e-08 | TGTGAACAGCAATAC |
| *X. tropicalis* | + | 4.57e-08 | TTGGAACAGCAATGC |
| *A. carolinensis* | + | 7.01e-08 | TTTGAACAGCAGGAC |
| *B. taurus* | + | 1.87e-07 | TGGAAACAGCAAAGC |
| *C. pyrrhogaster* | - | 4.21e-07 | TCTGAACAGTAAGGC |
| *M. musculus* | + | 7.76e-07 | TGTAAACACCAAGTC |
| *D. rerio* RPE65b | - | 1.62e-06 | TTTAAACAGAAATAC |
| *C. lupus familiaris* | - | 5.80e-06 | CGGGCACAGCTGGCC |
| MEME consensus sequences were analyzed using TOMTOM (an all vertebrate database) to compare function: Sandelin-Wasserman Similarity.  The corresponding accession numbers for these sequences are shown in S. Table 1 Online Resource. | | | |

| Supplementary Table 6. Conserved motif 5 description | | | | |
| --- | --- | --- | --- | --- |
| Motif 5  Uncharacterized  Tentative TF TOMTOM p<0.005:  Dbx1_3486.1 (Developing brain box)  Lhx2_DBD_2 | | | 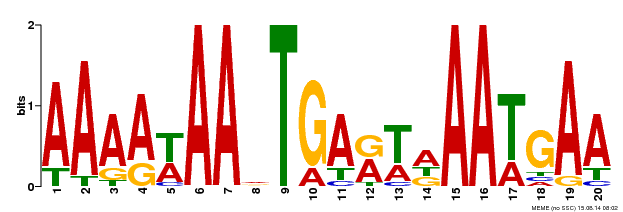 | |
| RPE65 promoter | **Strand** | ***p*-value** | | **Sites** |
| *H. sapiens* | + | 5.39e-11 | | AAAATAAGTGAGTTAATGAA |
| *G. gallus* | + | 1.64e-09 | | AAAATAAGTGTGAAAATGAA |
| *C. lupus familiaris* | + | 2.48e-09 | | AAAAAAAATGAAAAAAAGAA |
| *D. rerio* RPE65b | − | 2.46e-08 | | AAGGAAAATGAATGAATGAT |
| *C. picta bellii* | + | 3.25e-08 | | AAAATAAATAAATTAATGGA |
| *M. musculus* | + | 6.47e-08 | | TTAGTAATTGAGTAAATGAA |
| *X. tropicalis* | − | 1.50e-07 | | TAAAAAACTGAGTAAATTAT |
| *A. carolinensis* | + | 3.10e-07 | | AAAACAAATGAAATAATCAC |
| *B. taurus* | + | 3.50e-07 | | AAAATAAGTAATTGAAAAAA |
| *C. pyrrhogaster* | − | 6.13e-07 | | AATGTAACTGCACAAAAGAA |
| MEME consensus sequences were analyzed using TOMTOM (an all vertebrate database) to compare function: Sandelin-Wasserman Similarity.  The corresponding accession numbers for these sequences are shown in S. Table 1 Online Resource. | | | | |
